# Synthesis of 1α,25-Dihydroxyvitamin D Analogues Featuring a *S*_2_-symmetric CD-ring Core

**DOI:** 10.3390/molecules14020894

**Published:** 2009-02-24

**Authors:** Garrett Minne, Lieve Verlinden, Annemieke Verstuyf, Pierre J. De Clercq

**Affiliations:** 1 Department of Organic Chemistry, Ghent University, Krijgslaan 281, B-9000 Gent, Belgium; 2 Laboratory of Experimental Medicine and Endocrinology, Catholic University of Leuven, Gasthuisberg, Herestraat 49, B-3000 Leuven, Belgium

**Keywords:** Suzuki coupling, Sonogashira reaction, Calcitriol analogues.

## Abstract

Three analogues of 1α,25-dihydroxyvitamin D_3_ (calcitriol), featuring a *trans-*fused decalin C,D-core with local *S*_2_-symmetry, and possessing identical side-chain and seco-B,A-ring structures, have been synthesized starting from readily available (4a*R*,8a*S*)-octahydronaphthalene-1,5-dione (**7**). The very short sequences involve the simultaneous introduction of the side-chain and seco-B,A-ring fragments via Suzuki and Sonogashira coupling reactions. The analogues are devoid of relevant biological activity.

## Introduction

**Scheme 1:**
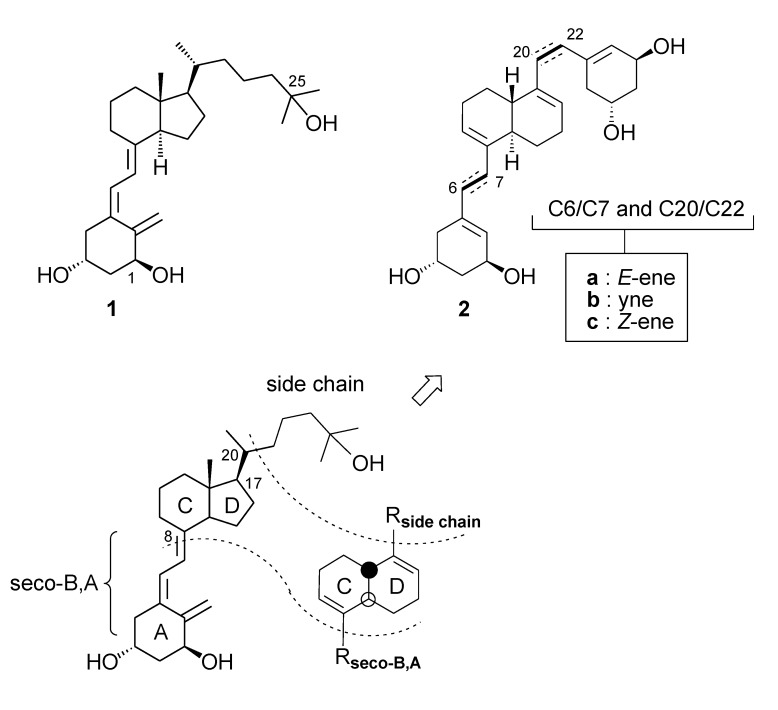
Calcitriol (**1**) and pseudo-symmetric analogues **2**.

Since the discovery that the biological action of vitamin D_3_ originates from the dihydroxylated metabolite 1α,25-dihydroxyvitamin D_3_ (**1**, calcitriol) and that, next to its classical role in the regulation of calcium homeostasis, these actions also involve immunomodulation, cell differentiation and antiproliferation, there has been an intense search for structural analogues of calcitriol that might show a separation in calcemic and antiproliferative-prodifferentiating activities (Scheme 1) [1]. In this context various successful structural modifications have been introduced in each one of the three parts that one may distinguish in its structure: the rigid central C,D-ring system and the flexible parts of the molecule, comprising the side chain and the seco-B,A-ring [2]. 

At the molecular level calcitriol generates biological responses via signal transduction pathways in which interaction with the nuclear receptor (n-VDR) leads to gene transcription regulation (genomic pathway) and in which interaction with a putative membrane receptor (m-VDR) leads to rapid actions such as transcaltachia (non-genomic pathway) [3]. A turning point in the rational development of analogues has been the disclosure of the detailed structure of the ligand binding domain of the n-VDR, obtained via X-ray diffraction analysis of the complex between calcitriol and a truncated mutant of the n-VDR [4]. Interestingly, this study revealed that the position of the ligand within the binding site was opposite to the location that was previously suggested on the basis of extensive molecular modeling studies [5]. This could indicate that the two polar parts of the molecule are interchangeable so that each bonding type could be associated with a different biological action. In this context we became interested in the development of analogues featuring structural symmetry so that the two flexible parts would become indistinguishable. Herein we wish to describe analogues **2a, 2b** and **2c** which are characterized by the presence of a *trans*-fused decalin C,D-core with local *S*_2_-symmetry (Scheme 1) [6].

## Results and Discussion

**Figure 1:**
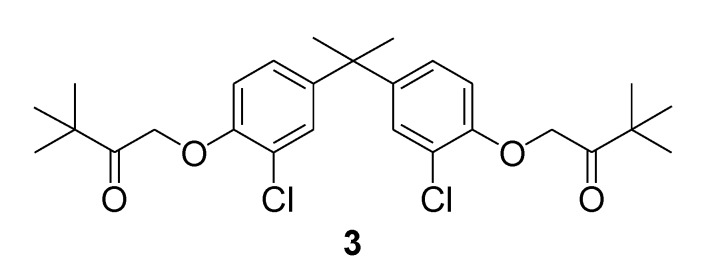
Non-steroidal analogue.

**Figure 2:**
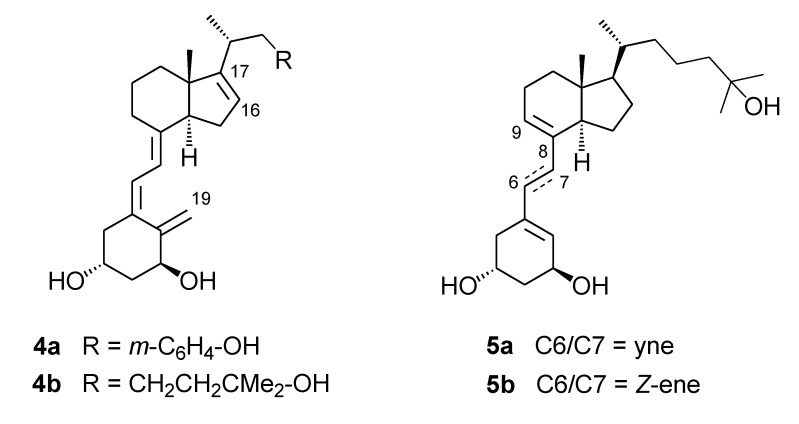
Structural modifications in calcitriol analogues.

The choice of a *S*_2_-symmetric *trans*-decalin core functionalized as in **2** was dictated both by structural and synthetic considerations. Indeed symmetrical non-steroidal derivatives have been described that were found to possess vitamin D-like activity (Figure 1). In particular **3** is able to induce VDR-mediated transactivation, albeit much less potently than calcitriol [7]. Also in the context of structural modifications the following are relevant to the present work (Figure 2): (i) cyclic motifs in the side chain (e.g. **4a**) [8,9]; (ii) unsaturation at C16,C17 (e.g. **4a**, **4b**) [10]; (iii) unsaturation at C8,C9 (e.g. **5a**, **5b**) [11]; and (iv) analogues featuring an enlarged six-membered D-ring [12].

In principle the structural features of analogues **2** should allow for very direct and convergent syntheses in which the two identical fragments representing the side chain and the seco-B,A-ring are introduced simultaneously on a suitable functionalized *trans*-decalin core in a process were stereoisomers would not be formed. This is in contrast with the often lengthy sequences and difficult separations that are otherwise required [13,14,15]. Finally, whereas most efforts in the development of analogues have been directed towards modifications in the flexible parts, our laboratories have always focused on the central less accessible part of the molecule, and almost as a rule, such modifications have led to a reduction in calcemic activity [16].

**Scheme 2:**
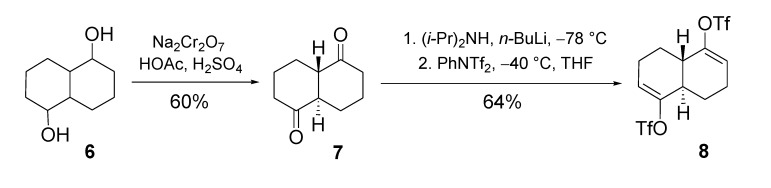
Synthesis of bis-enol triflate **8**.

The synthesis of the symmetrical analogues **2** proceeds via cross-coupling procedures involving the central bisenol triflate derivative **8**, which is readily obtained from the corresponding *trans*-fused diketone **7** (Scheme 2). The synthesis of the latter has been described in detail [17,18]. It involves the catalytic hydrogenation of 1,5-naphthalenediol over Raney Ni W-7, followed by sodium dichromate oxidation of the resulting mixture of stereoisomeric diols (**6**) in an acid medium [19], which affords, after purification by crystallization, diketone **7** in 40% overall yield. Kinetic deprotonation followed by treatment of the enolate dianion with *N*-phenyltrifluoromethane sulfonamide yielded crystalline **8**, which was purified by HPLC (64%). 

**Scheme 3:**
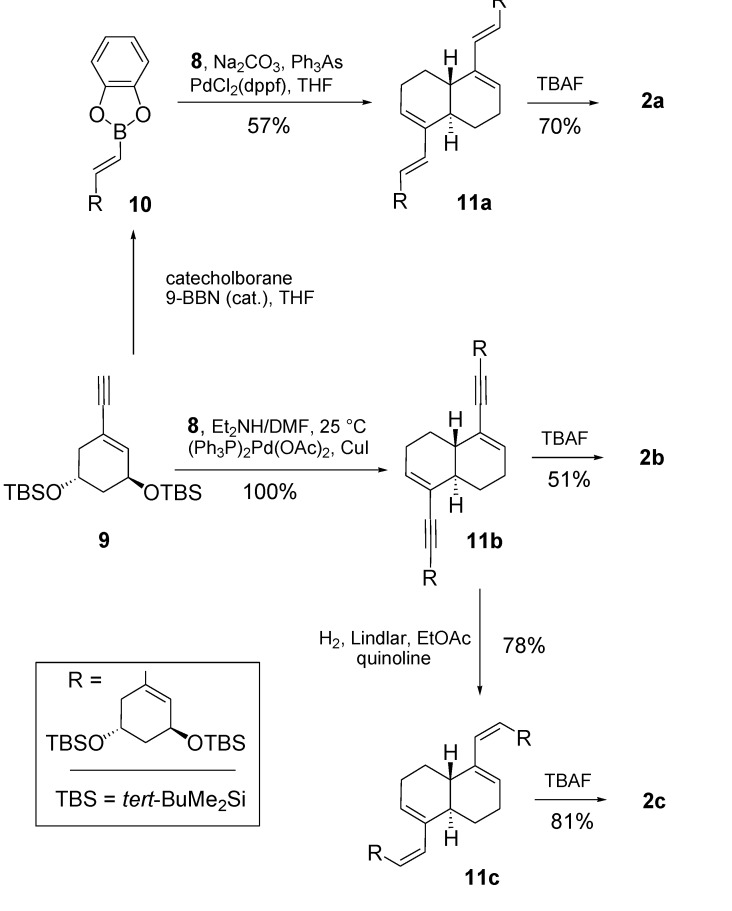
Synthesis of analogues **2a**, **2b** and **2c**.

The synthesis of analogues **2a**, **2b** and **2c** involves the known enyne **9** as central intermediate [11,20]. Depending on the nature of the C−C linkage at C17 and C8 either a Suzuki coupling (cf. **2a**) or Sonogashira coupling (cf. **2c**) is applied (Scheme 3) [21,22].

After extensive experimentation the Suzuki cross-coupling involving reaction of bisenol triflate **8** and intermediate **10**, which is obtained via addition of catecholborane to enyne **9** in the presence of 9-borabicyclononane (9-BBN) [23], afforded **11a** using a combination of 1,1’-bis(diphenylphosphino)ferrocene palladium(II)chloride (PdCl_2_(dppf)) with sodium carbonate as the base and the ligand triphenylarsine as reducing agent (57% yield) [24,25]. Deprotection with tetrabutylammonium fluoride (TBAF, THF) led to crystalline **2a** (70% yield).

The procedure developed by Castedo and Mouriño for coupling of a C-ring vinyl triflate with an A-ring terminal alkyne led to the desired **11b** in quantitative yield [26]. Semi-hydrogenation of the yne-moieties with Lindlar catalyst in the presence of quinoline gave **11c** in 78% yield. Eventual deprotection with TBAF yielded the crystalline analogues **2b** (51%) and **2c** (81%).

The biological evaluation of the analogues includes the determination of the binding affinity for the porcine intestinal VDR, and the *in vitro* antiproliferative activity on breast cancer MCF-7 cells [27]. The analogues **2a**, **2b** and **2c** did not show any relevant biological activity. This result shows that the side-chain fragment of the analogues in this series is probably too large.

## Conclusions

In the present work advantage is taken of the *S*_2_-symmetry of the readily available bisenol triflate **8** to introduce simultaneously fragments that may be considered as structural variations of the classical side-chain and of the seco-B,A-ring of calcitriol. The attachment of these fragments proceed in an efficient way via palladium catalyzed cross-couling processes involving either a Sonogashira reaction or a Suzuki coupling. Unfortunately the prepared calcitriol analogues **2a**, **2b** and **2c** were devoid of biological activity.

## Experimental

### General

TLC were run on glass plates precoated with silica gel (Merck, 60F-254). Column chromatography was performed on silica gel (Merck, 230-400 mesh) or Florisil (100-200 mesh). IR spectra were recorded on a Perkin-Elmer series 1600 FT-IR spectrometer. ^1^H-NMR and ^13^C-NMR spectra were recorded on a Bruker AM-500 spectrometer. Mass spectra (EI) were recorded on a Hewlett-Packard 5898A spectrometer at 70 eV.

*(4aR,8aS)-octahydronaphthalene-1,5-dione (**7**).* To a solution of decalin-1,5-diol **6** (8.86 g, 52 mmol) in benzene (70 mL) is slowly added dropwise the Cr(VI) oxidant over a period of 3 hours, during which period the temperature is kept at 6 °C. The oxidant is prepared separately by the successive addition to water (68 mL) of glacial acetic acid (11.5 mL), concentrated sulfuric acid (21 mL) and sodium dichromate dihydrate (15.51 g, 52 mmol). After stirring overnight the aqueous phase is separated and further extracted with toluene (3 x 25 mL). The combined organic phases are washed with water (25 mL), with a saturated solution of sodium bicarbonate (25 mL) and water again (25 mL). After concentration *in vacuo* the obtained residue is crystallized from benzene-petroleum ether (4:3) to yield 5.15 g of white crystalline diketone **7** (60% yield). R_f_ = 0.62 (dichloromethane/ethyl acetate, 9:1); m.p. 167-168 °C; IR (KBr): 2943 (s), 2908 (m), 2857 (m), 1702 (s), 1349 (m), 1261 (m), 564 (m), 488 cm^-1^; ^1^H-NMR (500 MHz, C_6_D_6_): δ = 2.10 (dddd, J = 13.6, 4.2, 2.1, 2.1 Hz, 2 H), 1.83 (dm, *J* = 13.8 Hz, 2 H), 1.65 (td, *J* = 13.7, 6.2 Hz, 2 H), 1.59-1.57 (m, 2 H), 1.55-1.49 (m, 2 H), 1.41-1.32 (m, 2 H), 1.07 (qt, *J* = 13.5, 4.1 Hz, 2 H) ppm; ^13^C-NMR (125 MHz, CDCl_3_): δ = 209.77 , 55.43 (CH), 41.13 (CH_2_), 24.92 (CH_2_), 24.36 (CH_2_) ppm; MS: m/z (%) = 166 (M^+^, 49), 138 (14), 123 (24), 110 (38), 98 (25), 97 (52), 95 (39), 84 (100), 83 (97), 68 (43), 67 (70), 55 (80).

*Bis-trifluoromethanesulfonate derived from (4aS,8aR)-3,4,4a,7,8,8a-hexahydronaphthalene-1,5-diol* (**8**). To a cooled (0 °C) solution of diisopropylamine (0.97 mL, 6.92 mmol) in THF is added dropwise a solution of *n*-butyllithium in hexane (4.43 mL of a 1.51 m solution, 6.92 mmol). After stirring for 1 hour a solution of diketone **7** (0.5 g, 3.01 mmol) in THF (20 mL) is added dropwise at −78 °C. After stirring for 5 hours at −78 °C *N*-phenyltrifluoromethane sulfonamide (2.364 g, 6.62 mmol) is added. After further stirring for 16 hours at −40 °C, the reaction mixture is brought to room temperature. After concentration *in vacuo* the residue is purified by column chromatography on silica gel with isooctane/ethyl acetate (9:1) as the eluent, followed by HPLC (95:5) to yield 822 mg of white crystalline bisenol triflate **8** (64%). R_f_ = 0.57 (isooctane/ethyl acetate, 9:1); m.p. 78-80 °C; IR (KBr): 2978 (m), 2956 (m), 2933 (m), 2877 (m), 1681 (m), 1415 (s), 1324 (m), 1252 (s), 1206 (s), 1143 (s), 1094 (s), 1051 (m), 935 (s), 870 (s), 626 (s), 604 (s) cm^-1^; ^1^H-NMR (500 MHz, CDCl_3_): δ = 5.79 (t, *J* = 2.4 Hz, 2 H), 2.59−2.52 (m, 2 H), 2.40 (ABm, *J* = 18.2 Hz, 2 H), 2.35−2.26 (m, 2 H), 2.22−2.18 (m, 2 H), 1.54−1.42 (m, 2 H) ppm; ^13^C-NMR (125 MHz, CDCl_3_): δ = 148.7 (C), 118.7 (CH), 118.6 (CF_3_, q, J_CF_ = 319 Hz), 41.8 (CH), 23.7 (CH_2_), 23.0 (CH_2_) ppm; MS: m/z (%) = 430 (M^+^, 3), 147 (28), 119 (20), 91 (55), 79 (20), 77 (29), 69 (100), 55 (81). 

*(4aR,8aS)-1,2,4a,5,6,8a-hexahydro-4,8-di((E)-2-((3S,5R)-3,5-dihydroxycyclohex-1-enyl)vinyl)naphtha-ene* (**2a**). To a solution of alkyne **9** (180 mg, 0.43 mmol) in THF (0.5 mL) is added dropwise a 1 m solution of catecholborane in THF (0.74 mL, 0.74 mmol). After stirring at room temperature for 15 min a 0.5 m solution of 9-borabicylo[3.3.1]nonane (9-BBN) in THF (125 μL, 0.063 μmol) is added. After stirring for 5 hours at 60 °C the reaction is brought to room temperature. To the red-orange solution is added via double-tipped needle a degassed solution of bisenol triflate **8** (50 mg, 0.116 mmol), 1,1’-bis(diphenylphosphino)ferrocene palladium(II) chloride (PdCl_2_(dppf), 9.8 mg, 0.012 mmol) and triphenylarsine (6 mg, 0.020 mmol) in THF, followed by a degassed 2 m sodium carbonate solution (0.4 mL, 0.81 mmol). After further stirring for 60 hours in the dark at room temperature the mixture is poured into water and extracted with methyl tert-butyl ether. After drying (magnesium sulfate) and concentration *in vacuo* the brown residue is purified by column chromatography (pentane/ethyl acetate, 98:2) to yield 57.1 mg of **11a** (57%) as an oil which is further subjected to deprotection.

To a solution of **11a** (210 mg, 0.242 mmol) in THF (2 mL) is added a 1 m solution of tetrabutylammonium fluoride in THF (2.0 mL, 2 mmol). After stirring overnight at room temperature the mixture is concentrated in vacuo. The brown residual oil is purified by repeated (2 times) column chromatography on silica gel (ethyl acetate/methanol, 92:8) to yield 70 mg of analogue **2a** as a white crystalline solid (70%). R_f_ = 0.34 (ethyl acetate/methanol, 9:1); m.p. 195 °C (decomposition); optical rotation: [α]_D_ = −86° (c = 0.07, methanol); IR (KBr): 3386 (s), 3330 (s), 2932 (s), 1620 (m), 1040 (s), 972 (s), 964 (s) cm^-1^; ^1^H-NMR (500 MHz, CD_3_OD): δ = 6.24 (s, 4 H), 5.83 (br s, 2 H), 5.73−5.72 (m, 2 H), 4.43−4.38 (m, 2 H), 4.16−4.11 (m, 2 H), 2.56 (dm, *J* = 16.7 Hz, 2 H), 2.31−2.17 (m, 8 H), 2.03 (ddm, *J* = 16.8, 7.1 Hz, 2 H), 1.88 (ABm, *J* = 13.1 Hz, 2 H), 1.82−1.76 (m, 2 H), 1.22−1.12 (m, 2 H) ppm; ^13^C-NMR (125 MHz, CD_3_OD): δ = 141.03 (C), 137.15 (C), 131.06 (CH), 129.87 (CH), 129.81 (CH), 129.45 (CH), 126.75 (CH), 126.66 (CH), 123.41 (CH), 66.03 (CH), 64.96 (CH), 42.95 (CH), 42.89 (CH), 40.70 (CH_2_), 34.59 (CH_2_), 28.10 (CH_2_), 27.82 (CH_2_) ppm; MS: m/z (%) = 374 (4), 356 (23), 338 (75), 179 (28), 167 (57), 154 (31), 141 (82), 128 (68), 115 (61), 91 (100), 86 (31), 49 (48), 45 (46).

*(4aR,8aS)-1,2,4a,5,6,8a-hexahydro-4,8-di((E)-2-((3S,5R)-3,5-dihydroxycyclohex-1-enyl)ethynyl)napht-halene* (**2b**). To a solution of bisenol triflate **8** (500 mg, 1.16 mmol) and enyne **9** (1.023 g, 2.79 mmol) in diethylamine/dimethyl formamide (1:1, 26 mL) are added copper(I) iodide (66 mg, 0.35 mmol) and bistriphenylphosphine palladium(II) acetate (87 mg, 0.12 mmol). The reaction mixture is further stirred at room temperature in the dark for 3 hours. The reaction mixture is poured into water and further extracted with diethyl ether. The combined organic phases are washed with brine and dried (sodium sulfate). After concentration *in vacuo* the residue is purified by column chromatography on silica gel with pentane/dichloromethane (7:3) as the eluent, followed by HPLC (isooctane/ethyl acetate, 95:5), to yield 1.0 g of white crystalline **11b** which is further subjected to deprotection.

To a solution of **11b** (100 mg, 0.12 mmol) in THF (3 mL) is added a 1 m solution of TBAF in THF (0.76 mL, 0.76 mmol). After stirring for 17 hours at room temperature the reaction mixture is directly eluted (ethyl acetate/methanol, 9:1) on a short column of silica gel. The residue obtained after concentration in vacuo is purified by crystallization (2 times) from methanol to yield 24 mg of white crystalline **2b** (51% yield). R_f_ = 0.59 (ethyl acetate/methanol, 8:2); m.p. 225 °C (decomposition); optical rotation: [α]_D_ = −52° (c = 0.05, methanol); IR (KBr): 3360 (s), 2929 (s), 1261 (s), 1086 (s), 1048 (s), 809 (s) cm ^-1^; ^1^H-NMR (500 MHz, DMSO-d_6_): δ = 6.07 (br s, 2 H), 5.94 (t, *J* = 1.8 Hz, 2 H), 4.74 (d, *J* = 5.8 Hz, 2 H), 4.64 (d, *J* = 4.0 Hz, 2 H), 4.24−4.18 (m, 2 H), 3.95−3.90 (m, 2 H), 2.30−2.22 (m, 8 H), 1.97−1.90 (m, 4 H), 1.67−1.59 (m, 4 H), 1.28−1.23 (m, 2 H) ppm; ^13^C-NMR (125 MHz, DMSO-d_6_): δ = 136.09 (CH), 135.45 (CH), 123.75 (C), 119.20 (C), 90.51 (C), 87.24 (C), 63.06 (CH), 62.05 (CH), 39.47 (CH), 38.90 (CH_2_), 38.21 (CH_2_), 26.29 (CH_2_), 25.96 (CH_2_); MS (APCI): m/z = 389 (2; M^+^-OH).

*(4aR,8aS)-1,2,4a,5,6,8a-hexahydro-4,8-di((Z)-2-((3S,5R)-3,5-dihydroxycyclohex-1-enyl)vinyl)naphtha-lene* (**2c**). To a solution of enyne **11b** (200 mg, 0.232 mmol) in ethyl acetate is added quinoline (3 mL of a 0.17 m solution in hexane, 0.232 mmol) and Lindlar catalyst (120 mg, purchased from Aldrich and dried for 3 hours *in vacuo*). The mixture is stirred under an atmosphere of hydrogen at room temperature and the reaction followed by TLC. After disappearance of starting material diethyl ether is added and the mixture filtered over Celite^®^. After concentration *in vacuo* the residue is purified by column chromatography on silica gel with isooctane/ethyl acetate (98:2) as the eluent and by HPLC with 0.2% methyl *tert*-butyl ether in isooctane as the eluent to yield 157 mg of **11c** as a colorless oil (78%) which is further subjected to deprotection.

To a solution of **11c** (143 mg, 0.17 mmol) in THF (7 mL) is added TBAF (1 mL of a 1 m solution in THF, 1 mmol) dropwise. After stirring for 24 hours at room temperature the reaction mixture is directly eluted (ethyl acetate/methanol, 9:1) on a column of silica gel to yield 55 mg of white crystalline **2c** (81%). R_f_ = 0.27 (ethyl acetate/methanol, 9:1); m.p. 135 °C (decomposition); optical rotation: [a]_D_ = −41° (c = 0.11, methanol); IR (KBr): 3362 (s), 2916 (s), 1654 (w), 1637 (w) cm ^-1^. ^1^H-NMR (500 MHz, DMSO-d_6_): δ = 5.91−5.80 (m, 4 H), 5.65 (m, 1 H), 5.63 (m, 1 H), 5.45 (m, 1 H), 5.41 (m, 1 H), 4.60 (d, *J* = 5.5 Hz, 1 H), 4.56 (d, *J* = 5.6 Hz, 1 H), 4.53 (d, *J* = 4.3 Hz, 1 H), 4.51 (d, *J* = 4.2 Hz, 1 H), 4.22−4.19 (m, 2 H), 3.87−3.79 (m, 2 H), 2.38 (ABd, *J* = 17.3, 4.5 Hz, 1 H), 2.25 (ABd, *J* = 17.1, 4.7 Hz, 1 H), 2.18−2.10 (m, 6 H), 1.99−1.91 (m, 3 H), 1.86 (ABd, *J* = 17.2, 7.5 Hz, 1 H), 1.70−1.64 (m; 2 H), 1.58−1.51 (m, 2 H), 1.23−1.15 (m, 2 H) ppm; ^13^C-NMR (125 MHz, DMSO-d_6_): δ = 138.55 (C), 138.49 (C), 134.73 (C), 134.55 (C), 131.63 (CH), 131.47 (CH), 131.05 (CH), 129.81 (CH), 129.72 (CH), 126.65 (CH), 126.28 (CH), 63.70 (CH), 62.68 (CH), 40.80 (CH), 40.72 (CH), 38.04 (CH_2_), 37.56 (CH_2_), 26.47 (CH_2_), 26.10 (CH_2_) ppm; MS: m/z (%) = 392 (7; M^+^ - 18), 141 (28), 129 (34), 128 (31), 117 (37), 114 (37), 105 (36), 91 (100), 78 (41), 43 (75), 40 (45). 
